# The hallmarks of COVID-19 disease

**DOI:** 10.1371/journal.ppat.1008536

**Published:** 2020-05-22

**Authors:** Daolin Tang, Paul Comish, Rui Kang

**Affiliations:** 1 The Third Affiliated Hospital, Guangzhou Medical University, Guangzhou, Guangdong, China; 2 Department of Surgery, UT Southwestern Medical Center, Dallas, Texas, United States of America; University of Alberta, CANADA

## Abstract

Severe acute respiratory syndrome coronavirus-2 (SARS-CoV-2) is a novel coronavirus that has caused a worldwide pandemic of the human respiratory illness COVID-19, resulting in a severe threat to public health and safety. Analysis of the genetic tree suggests that SARS-CoV-2 belongs to the same *Betacoronavirus* group as severe acute respiratory syndrome coronavirus (SARS-CoV) and Middle East respiratory syndrome coronavirus (MERS-CoV). Although the route for viral transmission remains a mystery, SARS-CoV-2 may have originated in an animal reservoir, likely that of bat. The clinical features of COVID-19, such as fever, cough, shortness of breath, and fatigue, are similar to those of many acute respiratory infections. There is currently no specific treatment for COVID-19, but antiviral therapy combined with supportive care is the main strategy. Here, we summarize recent progress in understanding the epidemiological, virological, and clinical characteristics of COVID-19 and discuss potential targets with existing drugs for the treatment of this emerging zoonotic disease.

## Introduction

Humans have suffered from lethal infectious diseases, including viral outbreaks, for a long time. Severe acute respiratory syndrome coronavirus-2 (SARS-CoV-2) is a newly identified virus that differs from severe acute respiratory syndrome coronavirus (SARS-CoV) and Middle East respiratory syndrome coronavirus (MERS-CoV) but can cause similar symptomology associated with pneumonia (Table 1) [1, 2]. This viral disease was named “COVID-19” by the World Health Organization (WHO) and was first recognized in Wuhan, Hubei Province, in China in December 2019 and may originate from eating wildlife, an established tradition in the oldest of human cultures. Subsequent to its introduction in Thailand, the virus has spread to more than 200 countries and territories. WHO declared this disease to be a public health emergency of international concern (Box 1), characterized as a pandemic.

**Table 1:** Main differences between COVID-19, SARS, and MERS.

	COVID-19	MERS	SARS
***Epidemiology***			
**Time of origin**	December 2019	June 2012	November 2002
**Place of origin**	Wuhan, China	Jeddah, Saudi Arabia	Fushan, China
**Has travel history**	Yes	Yes	Yes
**Confirmed cases**	84,305 (China)* 187,327 (Italy)* 843,937 (US)* 2,649,680 (global)*	2,494	8,096
**Death cases**	4,642 (5.50%, China)* 25,085 (13.39%, Italy)* 46,838 (5.54%, US)* 184,643 (6.96%, global)*	858 (34%)	744 (9.2%)
**Healthcare worker cases**	1,716 (2.03%, China)*	244 (9.8%)	1,870 (23.1%)
**Spread**	Animal to human, then human to human	Animal to human, then human to human	Animal to human, then human to human
**Main transmission**	Airborne, contact	Airborne, contact	Airborne, contact
**Patient-to-healthcare-worker transmission**	Yes	Yes	Yes
**Months of epidemic period**	N/A	>39	8
**Infection control risk**	High	High	High
**Current status**	Active	A few new cases	No new cases
**Incubation period (d)**	4–7	2–15	2–14
**Infectivity, basic reproductive number**	1.4–6.47	0.3–1.3	2.2–3.7
***Virology***			
**Natural host**	Bat	Bat	Bat
**Intermediate host**	Pangolins?	Camels	Civets
**Human host**	SARS-CoV-2	MERS-CoV	SARS-CoV
**Lineage**	Beta-CoV lineage B	Beta-CoV lineage C	Beta-CoV lineage B
**Genome size**	29.9 kb	30.1 kb	27.9 kb
**Receptor**	ACE2	DPP4	ACE2
***Clinical features***			
**Principal symptoms**	Fever, cough, fatigue, and shortness of breath	Fever, cough, fatigue, shortness of breath, and acute renal failure	Fever, cough, fatigue, and shortness of breath
**Lab tests**	Abnormal blood counts, abnormal coagulation, organ dysfunction, cytokine storm	Abnormal blood counts, abnormal coagulation, organ dysfunction, cytokine storm	Abnormal blood counts, abnormal coagulation, organ dysfunction, cytokine storm
**CT scans**	Bilateral patchy shadows or ground glass opacity in the lungs	Bilateral patchy shadows or ground glass opacity in the lungs	Bilateral patchy shadows or ground glass opacity in the lungs
**Severe cases**	Sepsis and septic shock	Sepsis and septic shock	Sepsis and septic shock
***Clinical management***			
**Principal approach**	Early supportive therapy and monitoring	Early supportive therapy and monitoring	Early supportive therapy and monitoring
**Specific treatment**	No	No	No
**Vaccine**	No	No	No

*Infected and death data as of April 23, 2020.

**Abbreviations:** ACE2, angiotensin I-converting enzyme 2; CoV, coronavirus; CT, computed tomography; DPP4, dipeptidyl peptidase 4; MERS-CoV, Middle East respiratory syndrome coronavirus; N/A, not applicable; SARS-CoV, severe acute respiratory syndrome coronavirus; SARS-CoV-2, severe acute respiratory syndrome coronavirus-2

**Box 1. Public health emergency of international concern**

A public health emergency of international concern (PHEIC) is a formal declaration by the emergency committee of WHO regarding an extraordinary event that will affect global health security and may require an international coordinated response. The PHEIC was first defined in the revised International Health Regulations (IHR) in 2005, which provides a framework for the handling of public health events. According to the IHR (2005), all member states of WHO have the duty to detect, access, report, and respond to public health emergencies that satisfy any 2 of the following 4 criteria: (1) Is the public health impact of the event serious? (2) Is the event unusual or unexpected? (3) Is there a significant risk of international spread? and (4) Is there a significant risk of international travel or trade restrictions? [110] Since the IHR (2005) came into force on June 15, 2007, WHO has announced “PHEIC” six times. They were for the H1N1 influenza pandemic in 2009, polio eradication in South Asia and Africa in 2014, the Ebola virus outbreak in West Africa in 2014, Zika virus outbreaks in Brazil and other countries in 2016, the Ebola outbreak in the Democratic Republic of Congo in 2018, and the new SARS-CoV-2 outbreak in China currently. The intent of declaring a PHEIC is to prevent or shorten the international spread of disease and avoid unnecessary interference with international dealings and trade as well as human rights restrictions.

The Art of War (“Sunzi Bingfa”), the famous ancient Chinese military treatise written by Sun Tzu, describes a series of strategies to win a war. It said, “Know yourself and know your enemy, and you will never be defeated.” This is also important in the current war on the invisible enemy SARS-CoV-2. Here, we summarize the hallmarks of COVID-19 in its epidemiology, virology, and clinical features and management and discuss potential targets to treat this emerging human respiratory disease.

### Epidemiology

**Fig 1:**
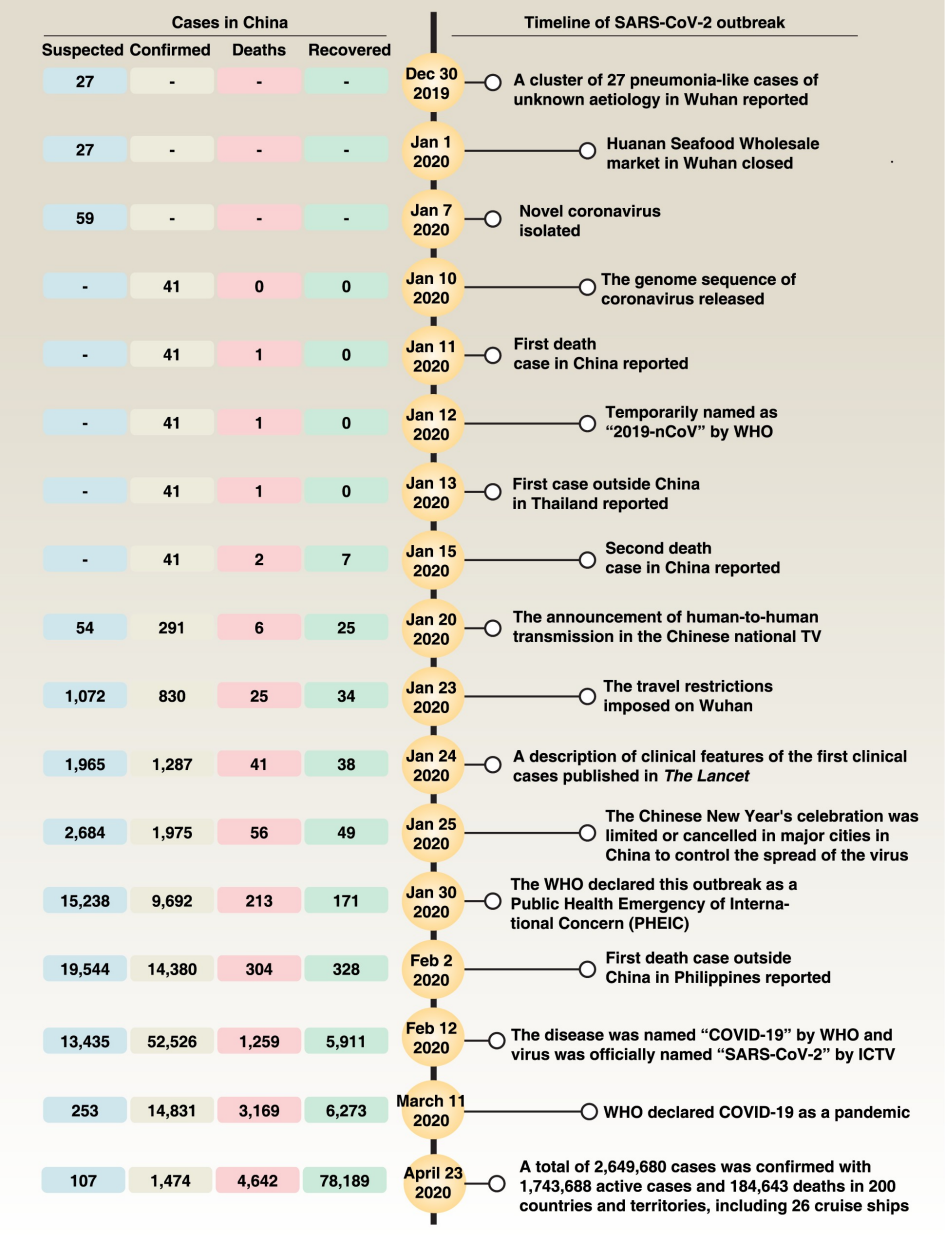
Main events of Wuhan coronavirus outbreak. ICTV, International Committee on Taxonomy of Viruses; SARS-CoV-2, severe acute respiratory syndrome coronavirus-2; WHO, World Health Organization.

On December 31, 2019, the Wuhan Municipal Health Committee first reported a cluster of 27 pneumonia-like cases of unknown etiology, including 7 severe cases, with a common reported link to the Huanan Seafood Wholesale Market in Wuhan (Fig 1) [2]. Later, a new strain of coronavirus was isolated from these patients, differing from SARS-CoV and MERS-CoV, albeit with some sequence similarity [2]. This virus was temporarily named “2019-nCoV” by WHO, and then officially named “SARS-CoV-2” by the International Committee on Taxonomy of Viruses (ICTV) [3].

Although important epidemiological risks include a history of travel from Wuhan or close contact with a patient with COVID-19 in the 14 days before symptom onset, recent studies argue that the Huanan Seafood Wholesale Market in Wuhan may not be the only source of SARS-CoV-2 infection, although 33 out of 585 samples taken from the market showed evidence of SARS-CoV-2. In fact, some early cases (8.6%–51%) have no epidemiological link with this market [4–8]. The main transmission route of SARS-CoV-2 from person to person is respiratory droplets or contact. Other possible routes include aerosol or oral-fecal transmission [9, 10]. Certain groups of the population, especially elderly men and those with underlying diseases, are more susceptible to SARS-CoV-2 infection [6, 11–13]. Children, infants, and pregnant women are also reported to have SARS-CoV-2 infection [14–16]. New evidence from Europe and the United States shows that young adults (between 20 and 54 years old) are also vulnerable to SARS-CoV-2 [17], which urges everyone to follow social distancing precautions. Based on the first 425 confirmed cases, the mean incubation period of the virus is 5.2 days, with a 95th percentile distribution of 12.5 days, and its basic reproductive number is 2.2, which is lower than the 3.0 for SARS-CoV [6]. More recently, 2 studies showed that the mean incubation period of the virus is 3 days (range, 0–24 days) or 4.75 days (range, 3–7.2 days), respectively [11, 18]. This survey discovered that only 1.18% of patients experienced a direct contact with wildlife, whereas 31.30% had been to Wuhan and 71.80% had contact with people from Wuhan [18], revealing the complex epidemiology of this outbreak. Notably, 4.5% patients with COVID-19 have no symptoms of pneumonia [11], highlighting the immense pressure for the early detection of SARS-CoV-2 infection, via laboratory testing. The basic reproductive number (R0)—the average number of secondary cases generated by a primary case—of SARS-CoV-2 is 1.4–6.47 [6, 19]. However, the R0 of SARS-CoV and MERS-CoV is 0.3–1.3 and 2.2–3.7, respectively, indicating that SRAS-CoV-2 may have a higher transmission capacity than SRAS-CoV and MERS-CoV [20]. As of April 23, 2020, a total of 2,649,680 cases were confirmed, with 1,743,688 active cases and 184,643 deaths in 200 countries and territories, including 26 cruise ships, which has put global public health institutions on high alert. Isolation and quarantine of infected individuals constitute the primary strategy for stopping or limiting the spread of disease.

### Virology

**Fig 2:**
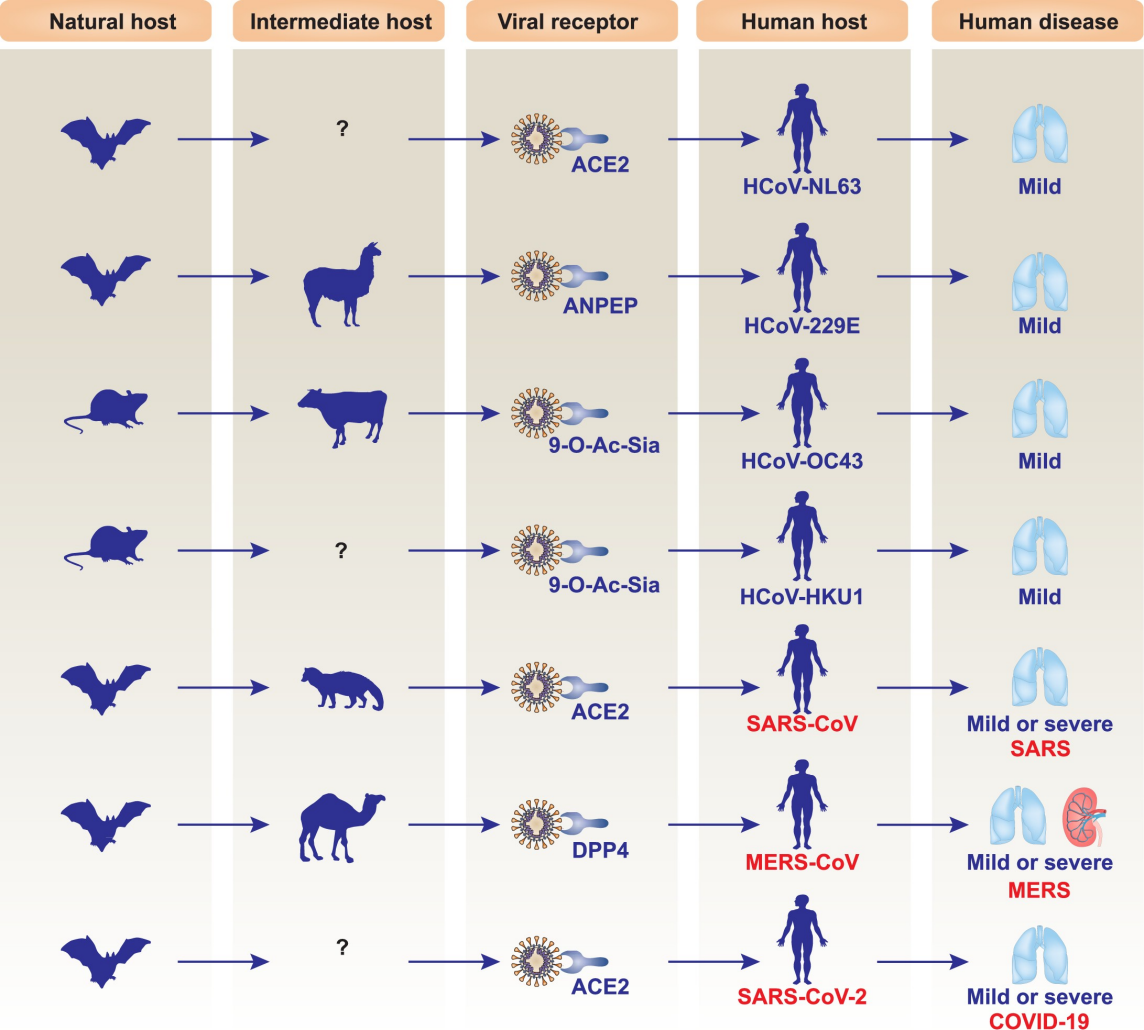
Hosts and consequences of human CoV infection. Different human CoVs have different natural and intermediate hosts. Among them, HCoV-229E, HCoV-OC43, HCoV-NL63, and HCoV-HKU1 cause mild infection, whereas SARS-CoV, MERS-CoV, and SARS-CoV-2 lead to mild or lethal respiratory diseases. 9-0-Ac-Sia, 9-O-acetylated sialic acids; ACE2, angiotensin I-converting enzyme 2; ANPEP (also known as CD13), alanyl aminopeptidase, membrane; CoV, coronavirus; DPP4 (also known as CD26), dipeptidyl peptidase 4; HCoV, human coronavirus; MERS-CoV, Middle East respiratory syndrome coronavirus; SARS-CoV, severe acute respiratory syndrome coronavirus; SARS-CoV-2, severe acute respiratory syndrome coronavirus-2.

Coronaviruses are enveloped, positive-sense, and single-stranded RNA viruses. They further divide into 4 subgroups, namely alpha, beta, gamma, and delta coronavirus. Several coronaviruses are zoonotic viruses that typically affect the respiratory and/or digestive tracts of mammals, including humans [21]. Since the first human coronavirus (HCoV) was discovered in the 1960s within the nares of patients with the common cold, 7 coronavirus species—including HCoV-229E, HCoV-OC43, HCoV-NL63, HCoV-HKU1, SARS-CoV, MERS-CoV, and SARS-CoV-2—have been discovered, leading to either mild or lethal respiratory disease depending on the strain type and host condition (Fig 2) [21]. Table 1 summarizes the main differences between SARS-CoV-2, SARS-CoV, and MERS-CoV and the diseases they cause. The size of the SARS-CoV-2 genome is 29.9 kb, while the genomes of SARS-CoV and MERS-CoV are 27.9 kb and 30.1 kb, respectively. Historically, SARS-CoV and MERS-CoV caused 8,096 and 2,494 cases, with mortality rates of 9.2% and 34%, respectively [2]. Currently, the SARS-CoV-2 mortality rate in China, Italy, the US, and the world is 4.01%, 12.63%, 2.98%, and 5.68%, respectively. Like other types of coronaviruses, isolated SARS-CoV-2 from clinical specimens has crown-like spikes seen on its surface using electron microscopy, with diameters varying from 60 to 140 nm [22]. The cytopathic effects induced by SARS-CoV-2 seem to be different from SARS-CoV and MERS-CoV. After SARS-CoV-2 invasion, structural changes in host cells are observed earlier in human airway epithelial cells (at 96 hours) than in other cell lines, including Vero E6 (at 144 hours) and Huh-7 (at 144 hours) [22].

**Fig 3:**
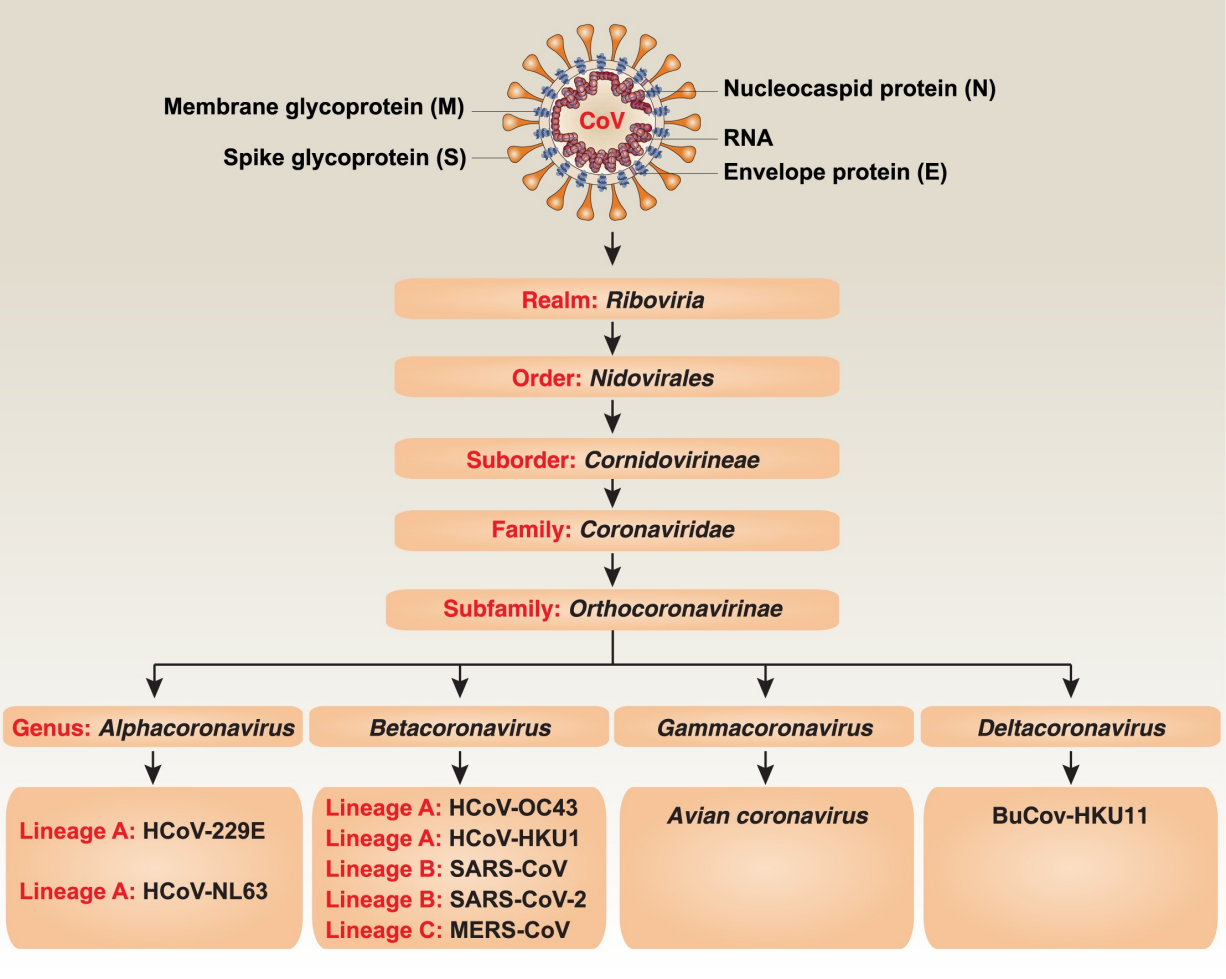
Schematic representation of the taxonomy of Coronaviridae. BuCoV-HKU11, bulbul coronavirus HKU11; HCoV, human coronavirus; MERS-CoV, Middle East respiratory syndrome coronavirus; SARS-CoV, severe acute respiratory syndrome coronavirus; SARS-CoV-2, severe acute respiratory syndrome coronavirus-2.

Obtaining the full genome of SARS-CoV-2 is a key to understanding its evolution and function. On January 10, 2020, the draft genome sequence of SARS-CoV-2 was first released on Virological.org [23]. One day later, 5 additional SARS-CoV-2 sequences, gathered from different patients, were deposited in the Global Initiative on Sharing All Influenza Data (GSAID) database, which is primarily used for sharing data on influenza viruses. Based on these shared data, genetic evolutionary analyses from different laboratories have shown that SARS-CoV-2 is a *Betacoronavirus* belonging to the *Sarbecovirus* subgenus of the Coronaviridae family, which is distinct from SARS-CoV (Fig 3) [22–27]. However, like SARS-CoV and MERS-CoV, bats may be the natural origin of SARS-CoV-2. SARS-CoV-2 has 86.9% to 96% nucleotide sequence similarity to multiple strains of bat SARS-like coronaviruses, such as ZC45, ZXC21, and RaTG3, which are on the same lineage (B) but are located on different branches [22, 24, 27]. It has been proposed that wild animals, such as civets and camels, further serve as the intermediate host for SARS-CoV and MERS-CoV, respectively [21]. The intermediate host required for SARS-CoV-2–mediated human disease is unknown. One early hypothesis is that snakes may be a bridge between bats and humans for SARS-CoV-2 infection [28], although there is no direct evidence that coronaviruses could adapt to cold-blooded hosts thus far. Recently, analysis of samples obtained from the Malytan pangolins in antismuggling operations from China showed that the pangolins are potential intermediate hosts for SARS-CoV-2, with 85.5% to 92.4% nucleotide identity to the SARS-CoV-2 genome [29, 30]. More recently, SARS-CoV-2 has been found to infect cats, ferrets, and tigers [31, 32]. However, it remains unknown what percentage of the same species of animal could be infected by SARS-CoV-2. It is also unclear how SARS-CoV-2 could jump from bats to pangolins or other animals.

The SARS-CoV-2 genome has 10 to 12 putative open reading frames (ORFs) [25, 33]. ORF1ab encodes nonstructural proteins (nsps), which are multifunctional proteins involved in virus processing and replication, while the remaining ORFs encode viral structural proteins (e.g., spike [S], envelope [E], membrane [M], and nucleocapsid [N]) and other accessory proteins (e.g., 3a, 3b, 6, 7a, 7b, 8, 9b, 9c, and 10). Notably, ORF1ab represents approximately 67% of the entire genome and encodes 15 or 16 nsps, depending on the bioinformatics analysis by different groups [25, 33]. One controversy is whether the tiny protein of nsp11 (4.8 kDa) exists alone and, if so, whether it plays a role in viral infections [25, 33].

**Fig 4:**
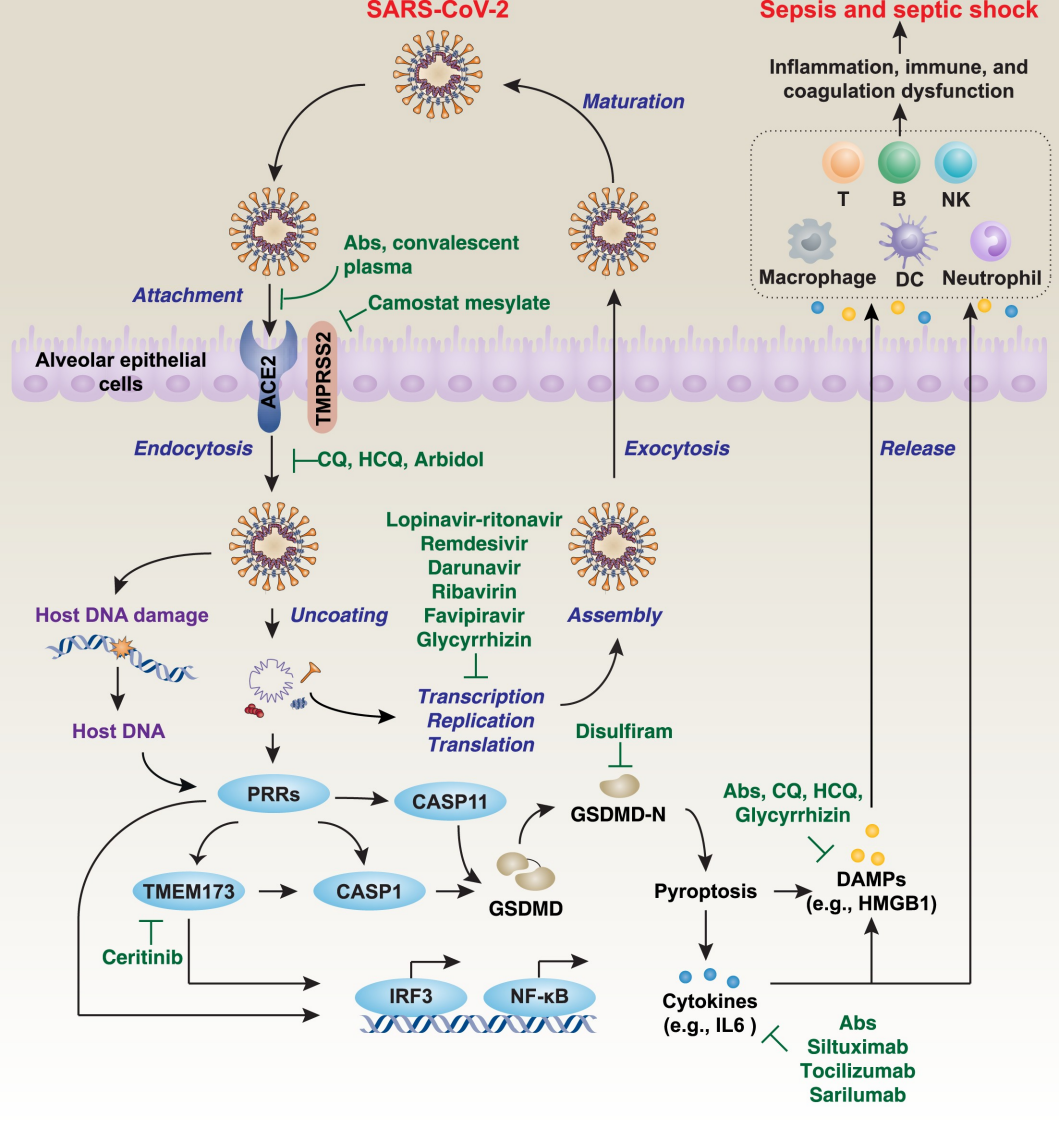
A model of the life cycle and immune response to SARS-CoV-2 in host cells. ACE2 is the host cell receptor responsible for mediating infection by SARS-CoV-2. After endocytosis and subsequent uncoating, the components of SARS-CoV-2 can be reused to produce new virus by host cells. Finally, the virus is released from the host cell by exocytosis. On the other hand, SARS-CoV-2–mediated host DNA damage or the components of SARS-CoV-2 can bind various cytosolic PRRs, leading to the activation of TMEM173- or GSDMD-dependent pyroptosis, which causes cytokine and DAMP release and subsequent inflammation, immunity, and coagulation dysfunction through impairment or activation of various immune cells, such as T cells, B cells, dendritic cells, NK cells, macrophages, and neutrophils. This process is involved in the activation of transcription factors, such as IRF3 and NF-κB. If not recognized early and managed promptly, it can lead to septic shock, multiple organ failure, and death. Ab, monoclonal antibody; ACE2, angiotensin I-converting enzyme 2; CASP1, caspase 1; CASP11, caspase 11; CQ, chloroquine; DAMP, damage-associated molecular pattern; DC, dendritic cell; GSDMD, gasdermin D; HCQ, hydroxychloroquine; HMGB1, high-mobility group Box 1; IRF3, interferon regulatory factor 3; NF-κB, nuclear factor κB; NK, natural killer; PRR, pattern-recognition receptor; SARS-CoV-2, severe acute respiratory syndrome coronavirus-2; TMEM173, transmembrane protein 173; TMPRSS2, transmembrane protease serine 2.

Structural proteins help the assembly and release of new copies of the virus. The M and E proteins are involved in the formation of the viral envelopes, while the N protein forms a helical ribonucleocapsid complex with positive-strand viral genomic RNA and interacts with viral membrane protein during assembly of virions [34]. The S protein is important for the attachment and entry of SARS-CoV-2 into host cells, causing syncytial formation between infected cells. During viral infection, the trimer S protein is cleaved into S1 and S2 subunits. The S1 subunit containing the receptor binding domain (RBD) is released during the transition to the postfusion conformation, whereas the membrane-anchored S2 subunit contains the fusion machinery. Angiotensin I-converting enzyme 2 (ACE2), especially expressed in type 2 alveolar epithelial cells, has been suggested as the cell entry receptor for SARS-CoV-2 into humans (Fig 4) [24, 27, 35]. In general, the SARS-CoV-2 first binds to ACE2 on the host cell surface through the S1 subunit and then fuses viral and host membranes through the S2 subunit. SARS-CoV also recognizes ACE2 as its receptor, whereas MERS-CoV recognizes dipeptidyl peptidase 4 (DPP4; also known as CD26) [21]. SARS-CoV-2 is more phylogenetically related to SARS-CoV than MERS-CoV [27]. It is worth noting that these receptors not only can serve as virus connection points but may also be important in virus entry, intracellular targeting, and uncoating.

Although the N and S proteins of SARS-CoV-2 are less conserved than other group 2B coronaviruses (SARS-CoV and MERS-CoV), the RBD in the S1 subunit of SARS-CoV-2 seems to be an important functional domain responsible for binding to the peptidase domain (PD) of the human ACE2 receptor protein [24, 25]. This is because several key residues of the RBD responsible for binding to the ACE2 receptor in SARS-CoV (e.g., Ala426, Thr487, Asn479, and Leu472) are changed in SARS-CoV-2 (e.g., Asn439, Asn501, Gln493, Gly485 and Phe486) [24]. However, the specific inhibitors or antibodies targeting these SARS-CoV-2 sites are still unidentified.

Most cryogenic electron microscopy (Cryo-EM) structural studies show that SARS-CoV-2 binds ACE2 with a higher affinity than SARS-CoV [36–38]. However, the biolayer interferometry study indicates that SARS-CoV-2 and SARS-CoV have similar affinity to ACE2 [39], indicating that other molecules may be involved in the modulation of ACE2-mediated SARS-CoV-2 invasion. The trimeric structure of the SARS-CoV-2 S protein includes one RBD in an “up” conformation and two RBD in a “down” conformation [36]. The “up” conformation of RBD of S protein binds to the PD of ACE2 with a *K*_*d*_ of about 15 nM [36]. Cryo-EM structural analysis further suggests that two S protein trimers can simultaneously bind to the ACE2 dimer mainly through polar residues [40]. Similar to SARS-CoV and MERS-CoV, the S protein trimer of SARS-CoV-2 occurs in many different conformational states [39], further indicating that different structures may independently have different virus invasion capabilities. In order for SARS-CoV-2 to enter the host cell, its S protein needs to be cleaved by cellular proteases at 2 sites, which is called S protein priming, so the viral and cellular membranes can fuse. SARS-CoV-2 can further use transmembrane protease serine 2 (TMPRSS2), a serine protease, to enter human lung cells by S protein priming, whereas the TMPRSS2 inhibitor camostat mesylate blocks cellular entry of the SARS-CoV-2 virus [41]. In addition to mediating S protein priming, TMPRSS2 may also participate in SARS-CoV-2 replication through unknown mechanisms [42]. In addition, the presence of 2 typical furin cleavage sites in the S protein of SARS-CoV-2 may play a similar role in promoting virus invasion and replication [39]. Because furin is abundant in the respiratory tract, the S protein may be cleaved after leaving the lung epithelial cells, thereby effectively infecting other cells. Therefore, the functional interaction between TMPRSS2 and furin is a key factor in determining the level of S protein priming. Other host proteins—such as heat shock protein family A (hsp70) member 5 (HSPA5, also known as GRP78) [43] and CD147 (an inducer of matrix metalloproteinase synthesis) [44]—may play an alternative or synergistic role in mediating SARS-CoV-2 invasion, although their structural basis is still unknown. It also remains to be seen whether ACE2 expression in other tissues, such as the gastrointestinal tract, kidney, and heart, has similar functions as in the lung.

Similar to SARS-CoV and MERS-CoV, the life cycle of SARS-CoV-2 is a dynamic process [34] (Fig 4). After membrane fusion, viral genomic RNA is released into the cytoplasm, while uncoated RNA translates 2 polyproteins, pp1a and pp1ab, which encode nsps and form a replication-transcription complex (RTC) in a double-membrane vesicle [34]. RTC continuously replicates and synthesizes a set of subgenomic RNAs that encode accessory and structural proteins [34]. After the components of RNA and protein are assembled, new viruses are produced and then released into the extracellular space of the host cell through exocytosis [34]. This information can be used to help develop antiviral drugs to suppress viral infections by disrupting the SARS-CoV-2 life cycle.

### Clinical features

The earliest study from the Jin-yin-tan Hospital in Wuhan described the clinical characteristics of the first 41 laboratory-confirmed COVID-19 patients, including 30 men and 11 women (median age of 49 years) [5] (Table 1). In total, 66% of patients had been exposed to the Huanan Seafood Wholesale Market, and 1 family cluster of SARS-CoV-2 infection was observed. In this cohort, 13 (31.7%) were admitted to an intensive care unit (ICU), and 6 (14.6%) died. Some patients had other health issues, such as diabetes (20%), hypertension (15%), cardiovascular disease (15%), chronic obstructive pulmonary disease (2%), chronic liver disease (2%), and cancer (2%). The clinical symptoms and signs were like many other acute respiratory infections, including SARS and MERS. With COVID-19, patients typically have fever (98%), cough (76%), dyspnea (55%; median time from illness onset to dyspnea of 8.0 days), and myalgia or fatigue (44%). Other signs, such as sputum production (28%), headache (8%), hemoptysis (5%), and diarrhea (3%), may be present. The median time from onset of symptoms to first hospital admission, shortness of breath, acute respiratory distress syndrome (ARDS), mechanical ventilation, and ICU admission was 7, 8, 9, 10.5, and 10.5 days, respectively.

Some COVID-19 patients had abnormal blood tests on admission, such as a decreased or normal white blood cell count, decreased lymphocyte count, prolonged prothrombin time, increased D-dimer level, or increased aspartate aminotransferase, creatinine, creatine kinase, and lactate dehydrogenase, indicating coagulation abnormalities and organ dysfunction. In contrast, the serum level of procalcitonin, a blood marker for bacterial infections, was normal in COVID-19 patients on admission.

Moreover, cytokine storms are associated with the development of SARS-CoV-2 infection. First, cytokines (e.g., IL1B, IL1RA, IL7, IL8, IL9, IL10, FGF, GCSF, GMCSF, IFNG, IP10, MCP1, MIP1A, MIP1B, PDGF, TNF, and VEGF) in plasma were significantly increased in patients with COVID-19 compared with the healthy control group. Second, certain pro-inflammatory cytokines (IL2, IL7, IL10, GCSF, IP10, MCP1, MIP1A, and TNF) were further increased in ICU patients compared with non-ICU patients, indicating that excessive acute inflammatory responses may lead to septic shock and death in COVID-19 patients. Another common abnormality was seen in chest computed tomography (CT) images (e.g., bilateral multiple lobular and subsegmental areas of consolidation), which was observed in 98% of COVID-19 patients.

A secondary study, also from the Jin-yin-tan Hospital in Wuhan, described the epidemiological and clinical characteristics of 99 laboratory-confirmed COVID-19 patients, including 67 men and 32 women (the median age of patients was 55.5 years) [7]. This study also suggests that the condemned market may not be the only source of the virus because only 49% of patients had a history of exposure to the Huanan Seafood Wholesale Market; 51% of patients had chronic diseases with impaired immune function, especially cardiovascular and cerebrovascular diseases (40%), endocrine system disease (13%), and digestive system disease (11%). On admission, most patients had fever (83%) or cough (82%), and one-third had shortness of breath (31%). Other symptoms included muscle aches (11%), headaches (8%), sore throat (5%), rhinorrhea (4%), chest pain (2%), and diarrhea (2%). The first 2 mortality cases demonstrated multiple organ failure and septic shock in a short period of time. T-lymphocyte injury may be an important factor that causes the patient’s condition to worsen. The low absolute value of lymphocytes can be used as a reference indicator for diagnosis. In addition to nasopharyngeal and oropharyngeal swabs, SARS-CoV-2 can sometimes be detected in stool samples as seen within the first case discovered in the US [45], raising the possibility of fecal-oral transmission.

More recently, updated studies based on laboratory-confirmed cases observed similar clinical, laboratory, and radiological features of the initial patients with COVID-19 on admission (Table 2) [8, 11, 18]. In general, fever, cough, and fatigue are the most common symptoms, although some patients display no symptoms. Bilateral patchy shadows or ground glass opacity in the lungs is the typical radiological finding on chest CT. Lymphopenia, thrombocytopenia, elevated C-reactive protein, up-regulated lactose dehydrogenase, increased D-dimer, and prolonged prothrombin time are the most common laboratory abnormalities, which are similar to those previously observed in patients with infection by MERS-CoV or SARS-CoV (Table 1). The disease severity is an independent predictor of a poor outcome. The first autopsy of a Chinese COVID-19 victim showed that the severity of pulmonary fibrosis and comorbidities was not as severe as SARS, and the exudation response was more pronounced than SARS [46]. Their alveoli were filled with fluid, white blood cells, mucus, and damaged lung cell debris [46]. There is no doubt that the lung is the most severely injured organ by SARS-CoV-2 infection; however, this virus can harm many other organs, such as the heart, liver, kidney, brain, and intestines. These clinical and laboratory findings provide important information on the diagnosis of SARS-CoV-2 infection, which is associated with immune dysfunction, altered coagulation, and tissue injury.

**Table 2:** Clinical, laboratory, and radiological features of COVID-19.

	Huang et al., 2020 [5]	Chen et al., 2020 [7]	Wang et al., 2020 [8]	Guan et al., 2020 [18]
**Total of laboratory-confirmed cases**	41	99	138	1,099
**Age, median, y**	49 (41–58)	55.5 (21–82)	56 (42–68)	47 (35–58)
**Male**	30 (73%)	67 (68%)	75 (54.3%)	640 (58.2%)
**Female**	11 (27%)	32 (32%)	63 (45.7%)	459 (41.8%)
**Huanan Seafood Wholesale Market exposure**	27 (66%)	49 (49%)	12 (8.7%)	N/A
**Local residents of Wuhan**	N/A	N/A	N/A	483 (43.9%)
**Wildlife exposure**	N/A	N/A	N/A	13 (1.2%)
**Nonlocal residents: Recently been to Wuhan**	N/A	N/A	N/A	193 (17.5%)
**Nonlocal residents: Contact with people from Wuhan**	N/A	N/A	N/A	442 (40.21%)
**Current smokers**	3 (7%)	N/A	N/A	137 (12.4%)
***Clinical features***				
**Any comorbidities**	13 (32%)	50 (51%)	64 (46.4%)	255 (23.2%)
**Hypertension**	6 (15%)	N/A	43 (31.2%)	164 (14.9%)
**Cardiovascular disease**	6 (15%)	40 (40%)	20 (14.5%)	27 (2.5%)
**Diabetes**	8 (20%)	13 (13%)	14 (10.1%)	81 (7.4%)
**Malignancy**	1 (2%)	1 (1%)	10 (7.2%)	10 (0.9%)
**Cerebrovascular disease**	N/A	1 (1%)	7 (5.1%)	15 (1.4%)
**Chronic respiratory system disease**	1 (2%)	1 (1%)	4 (2.9%)	12 (1.1%)
**Chronic kidney disease**	N/A	N/A	4 (2.9%)	8 (0.7%)
**Chronic liver disease**	1 (2%)	11 (11%)	4 (2.9%)	23 (2.1%)
**HIV infection**	N/A	N/A	2 (1.4%)	N/A
***Signs and symptoms***				
**Fever**	40 (98%)	82 (83%)	136 (98.6%)	473 (43.1%)
**Fatigue**	18 (44%)	N/A	96 (69.6%)	419 (38.1%)
**Cough**	31 (76%)	81 (82%)	82 (59.4%)	744 (67.7%)
**Anorexia**	N/A	N/A	55 (39.9%)	N/A
**Myalgia**	N/A	11 (11%)	48 (34.8%)	163 (14.8%)
**Dyspnea**	22 (55%)	31 (31%)	43 (31.2%)	204 (18.6%)
**Expectoration**	N/A	N/A	37 (26.8%)	367 (33.4%)
**Pharyngalgia**	N/A	5 (5%)	24 (17.4%)	153 (13.9%)
**Diarrhea**	1 (3%)	2 (2%)	14 (10.1%)	41 (3.7%)
**Nausea or vomiting**	N/A	1 (1%)	19 (14.7%)	55 (5.0%)
**Dizziness**	N/A	N/A	13 (9.4%)	N/A
**Headache**	3 (8%)	8 (8%)	9 (6.5%)	150 (13.6%)
**Abdominal pain**	N/A	N/A	3 (2.2%)	N/A
**Hemoptysis**	2 (5%)	N/A	N/A	10 (0.9%)
**ICU care**	13 (31.7%)	N/A	36 (26.1%)	55 (5%)
**Mortality**	6 (15%)	11 (11%)	6 (4.3%)	15 (1.36%)
***Laboratory features***				
**White blood cell count, ×10**^**9**^**/L**	6.2 (4.1–10.5); <4 (25%); >10 (30%)	7.5 (3.6); Increased (24%); Decreased (9%)	4.5 (3.3–6.2)	4.7 (3.5–6.0)
**Neutrophil count, ×10**^**9**^**/L**	5.0 (3.3–8.9)	5.0 (3.3–8.1); Increased (38%)	3.0 (2.0–4.9)	N/A
**Lymphocyte count, ×10**^**9**^**/L**	0.8 (0.6–1.1); <1 (63%); ≥1 (37%)	0.9 (0.5); Decreased (35%)	0.8 (0.6–1.1)	1.0 (0.7–1.3)
**Monocyte count, ×10**^**9**^**/L**	N/A	N/A	0.4 (0.3–0.5)	N/A
**Platelet count, ×10**^**9**^**/L**	164.5 (131.5–263.0) <100 (5%); ≥100 (95%)	213.5 (79.1); Increased (4%); Decreased (12%)	163 (123–191)	168.0 (132.0–207.0)
**Hemoglobin, g/L**	126.0 (118.0–140.0)	129.8 (14.8); Decreased (51%)	N/A	134.0 (119.0–148.0)
**Prothrombin time, s**	11.1 (10.1–12.4)	11.3 (1.9); Increased (5%); Decreased (30%)	13.0 (12.3–13.7)	N/A
**Activated partial thromboplastin time, s**	27.0 (24.2–34.1)	27.3 (10.2); Increased (6%); Decreased (16%)	31.4 (29.4–33.5)	N/A
**D-dimer, mg/L**	0.5 (0.3–1.3)	0.9 (0.5–2.8); Increased (36%)	203 (121–403)	≥0.5 (46.4%)
**Creatinine, μmol/L**	74.2 (57.5–85.7); ≤133 (90%); >133 (10%)	75.6 (25.0); Increased (3%); Decreased (21%)	72 (60–87)	≥133 (1.6%)
**Creatine kinase, U/L**	132.5 (62.0–219.0); ≤185 (68%); >185 (33%)	85.0 (51.0–184.0); Increased (13%); Decreased (23%)	92 (56–130)	≥ 200 (13.7%)
**Lactate dehydrogenase, U/L**	286.0 (242.0–408.0); ≤245 (28%); >245 (73%)	336.0 (260.0–447.0); Increased (7%)	261 (182–403)	≥250 (41.0%)
**Alanine aminotransferase, U/L**	32.0 (21.0–50.0)	39.0 (22.0–53.0); Increased (28%)	24 (16–40)	>40 (21.3%)
**Aspartate aminotransferase, U/L**	34.0 (26.0–48.0) ≤40 (63%); >40 (37%)	34.0 (26.0–48.0); Increased (35%)	31 (24–51)	>40 (22.2%)
**Albumin, g/L**	31.4 (28.9–36.0)	31.6 (4.0); Decreased (98%)		N/A
**Total bilirubin, mmol/L**	11.7 (9.5–13.9)	15.1 (7.3); Increased (18%)	9.8 (8.4–14.1)	>17.1 (10.5%)
**Blood urea nitrogen, mmol/L**	N/A	5.9 (2.6); Increased (6%); Decreased (17%)	4.4 (3.4–5.8)	N/A
**Hypersensitive troponin I, pg/mL**	3.4 (1.1–9.1); >28 (12%)	N/A	6.4 (2.8–18.5)	N/A
**Procalcitonin, ng/mL; ≥0.05**	12 (29.2%)	6 (6.6%)	49 (35.5%)	35 (5.5%)
**Sodium, mmol/L**	139.0 (137.0–140.0)	N/A	N/A	138.2 (136.1–140.3)
**Potassium, mmol/L**	4.2 (3.8–4.8)	N/A	N/A	3.8 (3.5–4.2)
**Chloride, mmol/L**	N/A	N/A	N/A	102.9 (99.7–105.6)
**Myoglobin, ng/mL**	N/A	49.5 (32.2–99.8); Increased (15%)	N/A	N/A
**Glucose, mmol/L**	N/A	7.4 (3.4); Increased (52%); Decreased (1%)	N/A	N/A
**C-reactive protein, mg/L**	N/A	51.4 (41.8); Increased (86%)	N/A	≥10 (60.7%)
**Serum ferritin, ng/mL**	N/A	808.7 (490.7); Increased (63%)	N/A	N/A
***Radiological features***				
**Bilateral distribution of patchy shadows or ground glass opacity on chest CT**	40 (98%)	88 (89%)	138 (100%)	840 (76.4%)

**Abbreviations:** CT, computed tomography; ICU, intensive care unit; N/A, not applicable

### Clinical management

SARS-CoV-2 infection can cause mild to severe illness, whereas severe infection can lead to ARDS, sepsis, septic shock, and even death. Guidelines for the clinical management of COVID-19 have been issued by WHO and each country, although the contents may be updated and enhanced over time. WHO-recommended management processes consist of: (1) screening and triage: early recognition of patients with severe acute respiratory infection associated with COVID-19; (2) immediate implementation of appropriate infection prevention and control (IPC) measures; (3) collection of specimens for laboratory diagnosis; (4) management of mild COVID-19: symptomatic treatment and monitoring; (5) management of severe COVID-19: oxygen therapy and monitoring; (6) management of severe COVID-19: treatment of coinfections; (7) management of critical COVID-19: ARDS; (8) management of critical illness and COVID-19: prevention of complications; (9) management of critical illness and COVID-19: septic shock; (10) adjunctive therapies for COVID-19: corticosteroids; (11) caring for pregnant women with COVID-19; (12) caring for infants and mothers with COVID-19: IPC and breastfeeding; (13) care for older persons with COVID-19; and (14) clinical research and specific anti–COVID-19 treatments [47]. These guidelines provide general principles for effective prevention, outbreak management, and disease treatment of SARS-CoV-2 infection in response to substantial advances in epidemiology, diagnostic methods, supportive care, and drug development. Notably, hospital-related transmission is a significant cause of COVID-19 infection and death in healthcare workers, which needs our utmost attention [8].

Because SARS-CoV-2 is an emerging virus, there are currently no specific drugs for treating diseases caused by its infection. So far, the main treatment is still supportive care, including increased oxygen delivery using a ventilator, fluid management, and antibiotic treatment. In addition, several antiviral drugs, human monoclonal antibodies, and other alternative medicines may be used only in the context of ethically approved clinical trials. For example, lopinavir-ritonavir, a drug that contains a combination of 2 medicines that have an anti-HIV effect, is being used to treat patients with COVID-19 in China [7]. However, a randomized clinical trial has shown that the benefits of treatment with lopinavir-ritonavir do not outweigh the benefits of standard treatment in patients with severe COVID-19 [48]. Remdesivir (GS-5734), a 1'-cyano-substituted adenosine nucleotide analog prodrug developed by Gilead Sciences Inc. (Foster City, CA) has shown efficacy in treating some patients with COVID-19 [45], but the results of ongoing randomized placebo-controlled trials remain unknown. Traditional Chinese medicine has a very long history in treating infectious diseases, although its treatments may have an unclear therapeutic mechanism. It is also possible that traditional Chinese medicine combined with Western medicines may improve symptoms, which is noted in the guidelines for the clinical management of COVID-19 in China [49]. Of note, several antiviral (e.g., oseltamivir), antibacterial (e.g., moxifloxacin, ceftriaxone, and azithromycin), and glucocorticoid therapies fail to provide significant benefit in treating patients with COVID-19 [8]. Thus, additional and improved therapies—including vaccines and new targeted therapy—are still urgently needed, and several clinical trials are underway. On March 16, 2020, the US started the first clinical trial of the COVID-19 candidate vaccine, which is mRNA-1273 (an mRNA vaccine against SARS-CoV-2 encoding for a prefusion stabilized form of the S protein) by Moderna and the Vaccine Research Center at the US National Institute of Allergy and Infectious Diseases. Other COVID-19 drugs or vaccines being developed by pharmaceutical companies around the world include TJM2 (a neutralizing antibody for human granulocyte-macrophage colony-stimulating factor [GM-CSF]) by I-Mab Biopharma (Shanghai, China), AT-100 (a recombinant form of human surfactant protein-D) by Airway Therapeutics (Cincinnati, OH), TZLS-501 (a neutralizing antibody for human IL6) by Tiziana Life Sciences (London, United Kingdom), BPI-002 (a small molecule agent to activate CD4+ helper T cells and CD8+ cytotoxic T cells) by BeyondSpring (New York, NY), INO-4800 (a vaccine) by Inovio Pharmaceuticals (San Diego, CA) and Beijing Advaccine Biotechnology (Beijing, China), TNX-1800 (a vaccine) by Tonix Pharmaceuticals (New York, NY), and recombinant subunit vaccine by Clover Biopharmaceuticals (Chengdu, China).

### Potential therapeutic targets

Although the pathogenesis of SARS-CoV-2 infection remains unclear, severe COVID-19 is a multiorgan dysfunction and life-threatening syndrome caused by a host response to the virus, which leads to an uncontrolled immune response and subsequent sepsis or septic shock through immediate and explosive release of various immune mediators, especially cytokines and damage-associated molecular patterns (DAMPs) [50] (Fig 4). Direct treatment strategies may include developing antibodies or inhibitors to block the interplay between S protein of SARS-CoV-2 and the host ACE2 receptor, generating oligonucleotides against the RNA genome of SARS-CoV-2, and administering passive antibodies from COVID-19 patients’ serum. Instead, drug repurposing may be a faster, more garish, and safer way for the evolution of treatments for COVID-19. In addition to antiviral drugs (e.g., remdesivir, penciclovir, galidesivir, and ribavirin), which are used in MERS or SARS as mentioned previously [51], we discuss some potential immunopathologic targets as well as related available drugs for the treatment of this new viral disease below.

### Host cell death

Most viral infections eventually lead to the death of host cells. Different types of regulated cell death (RCD) have distinct molecular mechanisms and signaling regulations (Box 2) [52]. Among them, pyroptosis is a widely studied form of pro-inflammatory cell death in immune cells as well as epithelial cells that is implicated in various infectious diseases, including viral infections [53, 54]. An excessive activation of pyroptosis mainly through inflammatory caspase 1 (CASP1) and caspase 11 (CASP11) (CASP4 and CASP5 in humans) can cause the cleavage of gasdermin D (GSDMD), which produces an N-terminal domain (GSDMD-N) to trigger cell death and the release of pro-inflammatory cytokines (e.g., IL1 and IL18) and DAMPs (e.g., high-mobility group Box 1 [HMGB1] and coagulation factor III [F3; also known as tissue factor]) [53–60]. This process is further modulated by many factors, such as Ca^2+^ influx, K^+^ efflux, and lipid peroxidation. GSDMD-N–mediated pyroptosis links canonical and noncanonical inflammasome activation to various immune responses and serves as a new target for the treatment of infectious diseases [61]. Indeed, GSDMD-deficient or -mutant mice are resistant to cecal ligation and puncture-induced septic shock or endotoxin lethality [53, 54, 57, 58, 60, 62, 63]. It is likely that a GSDMD inhibitor may limit virus-mediated host cell death. In particular, disulfiram, a drug used to treat alcohol addiction, strongly inhibits GSDMD function in vitro [64]. Because disulfiram has also been proven to be effective in inhibiting SARS-CoV, MERS-CoV, and HIV infection [65–67], it may also inhibit SARS-CoV-2 infection.

**Box 2. RCD**

Cell death is an important physiological or pathological phenomenon that is implicated in human health and diseases. There are many types of cell death, with classification based on different classification criteria. The oldest classification criteria was described by Schweichel and Merker and published in 1973 [111]. Accordingly, cell death is divided into type I (apoptosis), type II (autophagy-associated cell death), and type III (necrosis) cell death. Currently, the classification of cell death is switched from morphological criteria to molecular and genetic definitions from the Nomenclature Committee on Cell Death (NCCD) [112]. In general, cell death divides into accidental cell death (ACD) or RCD. ACD is a passive and uncontrolled process, whereas RCD is an active and controlled process. The main types of RCD include apoptosis, necroptosis, pyroptosis, ferroptosis, entotic cell death, netotic cell death, parthanatos, lysosome-dependent cell death, autophagy-dependent cell death, alkaliptosis, and oxeiptosis, which have a distinct molecular mechanism and signaling transduction. Apoptosis is usually triggered by the activation of caspases and further divides into extrinsic and intrinsic apoptosis, which are mainly mediated by CASP8 and CASP9, respectively [113]. Necroptosis is a mixed-lineage kinase domain-like pseudokinase (MLKL)-dependent regulated necrosis under the condition of caspase inhibition [114, 115]. Pyroptosis is mostly driven by GSDMD-N after the activation of CASP1 and CASP11 in response to extracellular or intracellular danger signals, including pathogen-associated molecular pattern molecules (PAMPs) and DAMPs [55–59]. Ferroptosis is a form of iron-dependent regulated necrosis, which requires the activation of lipid peroxidation [116, 117]. Parthanatos is a poly(ADP-ribose) polymerase 1 (PARP1)-dependent form of regulated necrosis [118], whereas entotic cell death is a type of cell-eat-cell death relying on the activation of entosis [119]. Netotic cell death [120], lysosome-dependent cell death [121], and autophagy-dependent cell death [122] are triggered by the activation of neutrophil extracellular traps, hydrolytic enzymes (e.g., cathepsins), or autophagy machinery (e.g., autophagy-related proteins), respectively. Alkaliptosis is mediated by intracellular alkalinization in cancer cells, whereas oxeiptosis is an oxygen radical-induced anti-inflammation form of cell death in immune cells.

### DAMPs

DAMPs are endogenous molecules that can be released or secreted by death stimuli or cytokines [68]. Most DAMPs are nuclear or cytosolic proteins, such as HMGB1, histones, the heat shock protein family, the S100 family, and mitochondrial transcription factor A (TFAM). Among them, HMGB1, the second most abundant nuclear protein, is a typical DAMP. Nuclear HMGB1 is an architectural chromatin-binding factor responsible for maintaining genome integrity, whereas extracellular HMGB1 is a mediator of inflammation and immune dysfunction in response to various stresses, including starvation, oxidative damage, hypoxia, and pathogen infection [69–72]. Thus, HMGB1 is an increasingly attractive target in various human diseases and pathologic conditions, especially critical illness and septic shock [73]. Given that HMGB1 is a potential target for SARS [74], we therefore hypothesize that HMGB1 may play a similar pathogenic role in COVID-19 by mediating inflammation and immune dysfunction. The pharmacological inhibition of HMGB1 release and activity by drugs (e.g., chloroquine and glycyrrhizin) has shown significant protective effects on lethal infection in mice [75]. Glycyrrhizin, a direct HMGB1 inhibitor, inhibits SARS-CoV replication [76]. Chloroquine, an aminoquinoline used for the prevention and therapy of malaria, is effective in protecting against sepsis and septic shock, partly through the inhibition of HMGB1 release and inflammation [77]. Chloroquine and its analogues (e.g., hydroxychloroquine) exhibit strong antiviral activity in preventing the replication and spread of SARS-CoV, MERS-CoV, and HIV through multiple mechanisms, such as increasing endosomal pH, hindering endosome fusion with lysosome, blocking ACE2 terminal glycosylation, or inhibiting S protein processing [78–84]. Chloroquine and hydroxychloroquine also inhibit SARS-CoV-2 production in culture cells in vitro [85–87]. Importantly, small clinical studies in China and France have shown that chloroquine and hydroxychloroquine are beneficial for the clinical efficacy and viral clearance of COVID-19 [88–90], and more research is ongoing globally. The antibiotic azithromycin further enhances this effect of hydroxychloroquine in some patients with COVID-19, indicating that a bacterial infection may worsen disease threats [90]. Randomized controlled trials are needed to determine the safety and efficacy of chloroquine in the treatment of COVID-19, because chloroquine may has a dual role in antivirus immunity [91].

### Transmembrane protein 173

Both PAMPs and DAMPs can function as a “signal 0” to initiate the innate immune response (Box 3) [68]. Thus, we reasoned that specialized pattern-recognition receptors (PRRs) may contribute to SARS-CoV-2–mediated immune dysregulation. In addition to well-known PRRs—such as toll-like receptors (TLRs), nucleotide-binding oligomerization domain (NOD)-like receptors, retinoic acid-inducible gene (RIG)-I–like receptors, absent in melanoma 2 (AIM2)-like receptors, C-type lectin receptors (CLRs), and advanced glycosylation end-product specific receptors (AGER/RAGE) [92]—we assume that transmembrane protein 173 (TMEM173; also known as STING), an intracellular immune regulator to PRR activation during infection and tissue injury [93, 94], may be implicated in SARS-CoV-2–mediated sepsis and septic shock. TMEM173 is activated by many stimuli, such as nuclear or mitochondrial DNA from host injury, nuclear acids from a DNA or RNA virus, or cyclic dinucleotide from gram-negative and gram-positive bacteria [93, 94]. Like other pathways of innate immune responses, the activation of the TMEM173 pathway may be the first line of defense against invading pathogens, including viruses [95–98]. However, the excessive activation of TMEM173 can produce type I interferon (IFNα and IFNβ) and various cytokines (e.g., IL6 and TNF), which cause a cytokine storm [99–105]. The excessive activation of TMEM173 also triggers inflammasome activation [106], GSDMD-N–mediated pyroptosis and subsequent lethal immunocoagulation response in experimental septic shock [60]. Notably, bat is resistant to various viral infections partly due to the loss of TMEM173-mediated type I interferon production [107]. In contrast, an excess of type I interferon drives SARS-CoV–induced lung injury and immune dysfunction in mice [108]. These findings make TMEM173 a potential target in SARS as well as COVID-19. Given that the drug ceritinib, an anaplastic lymphoma kinase (ALK) inhibitor, exhibits a promising role in protecting against experimental septic death through blocking the TMEM173 pathway [109], it remains interesting to see whether ceritinib can be used to suppress SARS-CoV-2–mediated organ dysfunction.

**Box 3. PAMPs and DAMPs**

The most important function of the immune system is its ability to distinguish various exogenous or endogenous danger signals, namely signal 0’s [68]. The exogenous danger signals referred to as PAMPs are components of microbes (e.g., bacteria, fungi, viruses, and parasites) including DNA, RNA, protein, and membrane components (e.g., lipopolysaccharide) [123]. In contrast, the endogenous danger signals are DAMPs, which are components of a host cell [124, 125]. DAMPs can be further divided into protein and nonprotein subgroups. The important sources of DAMPs are nuclear (e.g., HMGB1 and histone), cytosolic (e.g., heat shock proteins or the S100 family), or mitochondrial (e.g., TFAM, a structural and functional homolog of HMGB1) proteins. In addition, nonprotein DAMPs mainly include host DNA (e.g., genomic or mitochondrial DNA), host RNA (e.g., microRNA [miRNA]), adenosine triphosphate, and uric acid. DAMPs are not only passively released from dead or dying cells but also actively secreted by various immune cells, mainly through lysosome-dependent pathways. Both PAMPs and DAMPs can be recognized by the same or different PRRs, such as TLRs, NOD-like receptors, RIG-I–like receptors, AIM2-like receptors, CLRs, and AGER/RAGE, on cell membranes or within the cytosol, leading to inflammation. Although the appropriate inflammatory response is a defense mechanism to protect host cells from infection and injury, excessive or uncontrolled inflammation contributes to infection, tissue injury, and autoimmunity, which drives the pathogenesis of many human diseases. It is therefore critical to monitor the immune response to PAMPs or DAMPs.

### Conclusions and perspectives

SARS-CoV-2 is the third highly pathogenic HCoV discovered, which was first reported in Wuhan and has been rapidly spreading in China and beyond. As a global health concern, SARS-CoV-2 is more contagious, but less deadly, than SARS-CoV thus far. While bats have been implicated as the original hosts for SARS-CoV-2, its intermediate hosts as well as transmission routes among humans remain largely unclear. This novel coronavirus appears to use the same cell entry receptor—ACE2—as HCoV-NL63 and SARS-CoV, albeit with disparate disease severities (Fig 2). Since viruses are continually changing as a result of genetic selection, it is likely that SARS-CoV-2 will further adapt to the human host through mutations or recombination. Obtaining epidemiological information (e.g., contact history) and molecular diagnostic profiles of either animals or humans with SARS-CoV-2 can help us in understanding SARS-CoV-2 evolution and in developing preventive strategies or clinical interventions. Moreover, a large population-based cohort study of COVID-19 is needed to further determine molecular evidence of interhuman transmission and the disease’s clinical features, information that should be shared internationally. Most individuals with mild cases may recover fully without treatment, but those with severe cases definitively need ICU care. Drug repurposing may be an emerging option against COVID-19 because common molecular pathways contribute to many different pathogenic infections. Randomized controlled trials in patients on a large scale are required to evaluate the safety and efficacy of potential drugs in the treatment of SARS-CoV-2 infection. Finally, the long-term psychosocial impact of this epidemic on individual, national, and international levels remains to be evaluated after the end of this war on SARS-CoV-2.
